# Targeting Neurological Manifestations of Coronaviruses by Candidate Phytochemicals: A Mechanistic Approach

**DOI:** 10.3389/fphar.2020.621099

**Published:** 2021-01-20

**Authors:** Sajad Fakhri, Sana Piri, Mohammad Bagher Majnooni, Mohammad Hosein Farzaei, Javier Echeverría

**Affiliations:** ^1^ Pharmaceutical Sciences Research Center, Health Institute, Kermanshah University of Medical Sciences, Kermanshah, Iran; ^2^ Student Research Committee, Kermanshah University of Medical Sciences, Kermanshah, Iran; ^3^ Departamento de Ciencias del Ambiente, Facultad de Química y Biología, Universidad de Santiago de Chile, Santiago, Chile

**Keywords:** coronaviruses, COVID-19, SARS-CoV-2, neurology, nervous system, phytochemicals, pharmacology, signaling pathways

## Abstract

The novel coronavirus 2019 (COVID-19) caused by severe acute respiratory syndrome coronavirus 2 (SARS-CoV-2) has made a wide range of manifestations. In this regard, growing evidence is focusing on COVID-19 neurological associations; however, there is a lack of established pathophysiological mechanisms and related treatments. Accordingly, a comprehensive review was conducted, using electronic databases, including PubMed, Scopus, Web of Science, and Cochrane, along with the author’s expertize in COVID-19 associated neuronal signaling pathways. Besides, potential phytochemicals have been provided against neurological signs of COVID-19. Considering a high homology among SARS-CoV, Middle East Respiratory Syndrome and SARS-CoV-2, revealing their precise pathophysiological mechanisms seems to pave the road for the treatment of COVID-19 neural manifestations. There is a complex pathophysiological mechanism behind central manifestations of COVID-19, including pain, hypo/anosmia, delirium, impaired consciousness, pyramidal signs, and ischemic stroke. Among those dysregulated neuronal mechanisms, neuroinflammation, angiotensin-converting enzyme 2 (ACE2)/spike proteins, RNA-dependent RNA polymerase and protease are of special attention. So, employing multi-target therapeutic agents with considerable safety and efficacy seems to show a bright future in fighting COVID-19 neurological manifestations. Nowadays, natural secondary metabolites are highlighted as potential multi-target phytochemicals in combating several complications of COVID-19. In this review, central pathophysiological mechanisms and therapeutic targets of SARS-CoV-2 has been provided. Besides, in terms of pharmacological mechanisms, phytochemicals have been introduced as potential multi-target agents in combating COVID-19 central nervous system complications.

## Introduction

Phylogenetic studies on the genomic structure, introduced various types of coronaviruses (CoVs), including NL63, 229E, OC43, HKU1, middle east respiratory syndrome (MERS)-CoV, severe acute respiratory syndrome coronavirus (SARS-CoV), and severe acute respiratory syndrome coronavirus 2 (SARS-CoV-2) (Gurung et al., 2020a; Vellingiri et al., 2020), divided into four groups of alpha (229E and NL63), beta (OC43 and HKU1), gamma and delta coronaviruses. Among coronaviruses, alpha and beta groups cause respiratory manifestations in human (Gurung et al., 2020b; Gurung et al., 2020c; Rasool et al., 2020). Recently, a new strain of coronaviruses, namely SARS-CoV-2 has been found, belonging to a distinct class of beta coronaviruses (Divani et al., 2020). SARS-CoV-2 made a deadly disease, termed coronavirus disease 2019 (COVID-19) with devastating manifestation all over the world (Fitriani et al., 2020; Nemoto et al., 2020). The large and positive sense RNA genome with a size of 27–32 kb, as well as an envelope with spike (S1 and S2)/conjugated proteins (Holmes and Lai, 1996; Davidson et al., 2020) are associated with COVID-19 symptoms over a period of 2–14 days. Studies have revealed that when viruses enter to the lung tissue cells and proliferate, cause alveolar and interstitial inflammatory secretion and edema that leads to alveolar gas exchange impairment and hypoxia in the central nervous system (CNS), thereby increases anaerobic metabolism in the mitochondria of brain cells (Wu et al., 2020c). Besides, SARS-CoV enters the nasal passage and triggers neural inflammatory responses through dysregulation of the immune system. The entry factors for SARS-CoV-2 are highly expressed in nasal epithelial cells (Sungnak et al., 2020). As a consequence, CoVs enters the brain via the olfactory tract in the early stages of nasal vaccination or infection (Mori, 2015; Wu et al., 2020c; Desforges et al., 2020). Accordingly, this virus is not limited to the respiratory system but invades peripheral nerves and enters the CNS then causes/aggravates neurodegenerative disorders (Matsuda et al., 2004; Vellingiri et al., 2020). Research has shown the presence of SARS-CoV in cortex, hippocampus, spinal cord, brain stem, cerebellum, striatum, colliculus superior, and hypothalamus (Jacomy and Talbot, 2003). Consequently, COVID-19 patients have shown neurological symptoms, including headache, dizziness, hypogeusia, nausea, vomiting, and anosmia (Ahmad and Rathore, 2020; Vellingiri et al., 2020).

From the pathophysiological point of view, the spike protein in the morphology of COVID-19 bind to angiotensin-converting enzyme (ACE)-receptors on alveolar epithelial cell type 2 (AT2), primed by transmembrane protease serine 2 (TMPRSS2) to allow coronavirus entry (Marchetti et al., 2018; Li et al., 2019b; Wang et al., 2020b; Vallamkondu et al., 2020). Experimental evidence indicated that COVID-19 enters the lung via the respiratory tract and invades AT2 cells to generate a surfactant regarding declining related tension within alveoli to alleviate collapsing pressure. Also, ACE2 is presented in kidney, heart, enterocytes, pancreas, endothelial cells and widely distributed in brain to facilitate the SARS-CoV-2 entry into the cells (Li et al., 2003; Diao et al., 2020). The neural distribution of ACE2 was controversial at first. While a quantitative real-time RT-PCR study showed low levels of ACE2 mRNA in the human brain (Harmer et al., 2002), immunohistochemistry results indicated that ACE2 protein expression was restricted to arterial and endothelial smooth muscle cells (Hamming et al., 2004). Additionally, the predominant expression of ACE2 in glial cells was shown in brain primary cell cultures (Gallagher et al., 2006). Complementary evidence showed the widespread presence of ACE2 mRNA and protein throughout the brain (Doobay et al., 2007) or brainstem (Lin et al., 2008). Finding SARS-CoV in brains of infected patients also confirmed related distribution of ACE2 (Ding et al., 2004; Gu et al., 2005; Xu et al., 2005; Xia and Lazartigues, 2008).

As a critical sign of COVID-19, neuroinflammation occurs through elevated levels of neuronal interleukin (IL)-1β, IL-2, IL-4, IL-6, IL-8, IL-10, IL-12, tumor necrosis factor-α (TNF-α), interferon-gamma (IFN-γ), granulocyte colony-stimulating factor (GMCSF), IFN-γ-induced protein 10 (IP-10), monocyte chemoattractant protein-1 (MCP1), macrophage inflammatory protein 1α (MIP1α), and T cell expression (Xu et al., 2005; Yarmohammadi et al., 2020). The coronaviruses release inflammatory mediators to stimulate macrophages. These macrophages activate IL-1, IL-6, TNF-α, C-X-C motif chemokine ligand 10 (CXCL10) and chemokine ligand 2 (CCL2). Prevailing evidence is showing that CoVs reach the neurons, astrocytes, and/or microglia in CNS. Consequently, microglia and astrocytes play major roles in neuroinflammation and released inflammatory mediators (Murta et al., 2020). These cytokines and chemokines causes vasodilation and also increased capillary penetrance that causes declined surfactant stage in AP-2 cells which in turn lead to alveolar collapse and perturbation in gaseous exchange (Zaki et al., 2012; Wu et al., 2020a; Guan et al., 2020; Yang et al., 2020). In the other level of disease there is an increased level of inflammatory mediators via CD4^+^ and also increased generation of neutrophils and macrophages using IL-17, IL-21, and IL-22 that causes difficulties in breathing, hypoxemia, and cough (de Wit et al., 2016; Gao et al., 2020; Wan et al., 2020). In addition to the elevation of neuronal inflammatory mediators in CNS and related neuronal associations, ACE2/spike proteins and downstream mediators, RNA-dependent RNA polymerase (RdRP)/proteases, seem to be golden targets in stopping related neuronal signs.

Unfortunately, up to now there is no antiviral drug or vaccine for the treatment of coronaviruses infection, although candidate phytochemicals can be promising factors with antiviral potentials for the treatment of infection (Liu and Du, 2012; Gurung et al., 2020b). Some previous research has indicated neurological manifestations in coronaviruses (Ahmed et al., 2020; Yavarpour-Bali and Ghasemi-Kasman, 2020). Besides, limited studies suggested natural products and candidate phytochemicals as helpful agents for the prevention and treatment of coronaviruses (Hasan et al., 2020; Majnooni et al., 2020; Zhou and Huang, 2020). In this study, an extensive review was performed on neurological manifestations of coronaviruses, as well as the effects of candidate phytochemicals on the aforementioned signaling pathways. Additionally, this is the first review on highlighting phytochemicals with antiviral and neuroprotective effects, which targets the neural pathogenic pathways of CoVs (termed candidate phytochemicals) regarding the prevention and treatment of COVID-19 neuronal signs.

## Study Design

We used electronic databases (e.g., Scopus, PubMed, Medline, and Web of Science) and related articles in other sources, to conduct a comprehensive review on the neurological manifests of coronaviruses, as well as the phytochemicals effects. The keywords (“Severe Acute Respiratory Syndrome” OR “SARS” OR “Middle East Respiratory Syndrome” OR “MERS” OR “Coronavirus disease 2019” OR “COVID-19” OR “SARS-CoV” OR “SARS-CoV-2”) AND (“neurological sign” OR “neurological manifestation” OR “neuron” OR “nerve” OR “central nervous system” OR “CNS” OR “brain” OR “neurology” OR “neuropathy” OR “stroke” OR “multiple sclerosis” OR “encephalitis” OR “encephalopathy”) [title/abstract/keywords] were used. All the phytochemicals possessing both the antiviral and neuroprotective activities with the keywords (“phytochemical” OR “secondary metabolite” OR “plant” OR “polyphenol” OR “phenolic compound” OR “flavonoid” OR “alkaloid” OR “terpen” OR “terpenoid” OR “quinone”) were also searched in the whole text. Overall, the phytochemicals with reported antiviral and neuroprotective effects possessing the potential of modulating coronaviruses pathophysiological mechanisms were included. Data were collected without language and date restrictions until October 2020. The screening of retrieved articles was also done. on the reference lists/citation. Regarding completing review on the electronic databases, hand searching also was done relying on the authors' expertize on the SARS-CoVs pathophysiological mechanisms in CNS and candidate phytochemicals.

## Neuronal Manifestations of Coronaviruses

Experimental evidence showed two types of neurological manifestations referring to the CNS and peripheral nervous system (PNS). Of the PNS, there are various neurological associations such as hypogeusia, hyposmia, impaired eye movement, trigeminal neuropathy, Miller-Fisher syndrome, polyneuritis cranialis, rhabdomyolysis, Guillain-Barré Syndrome, and olfactory dysfunction (Ahmad and Rathore, 2020; de Freitas Ferreira et al., 2020; Mochan and Modi, 2020; Nordvig et al., 2020; Pascual-Goñi et al., 2020; Yavarpour-Bali and Ghasemi-Kasman, 2020). COVID-19 also causes CNS impairment such as cerebrovascular disorders, acute ischemic stroke (1–3%), intracranial haemorrhage (0.5%), encephalitis (brain inflammation), demyelination, meningitis, polyneuritis cranialis, vasculitis, and skeletal muscular damage (Li et al., 2016; Dorche et al., 2020; Filatov et al., 2020; Mao et al., 2020; Moriguchi et al., 2020; Zhou et al., 2020). It has been shown that 229E and OC43 coronavirus strains invade to neuroblastoma, neuroglioma, astrocytoma, microglial, and oligodendrocytic cell cultures (Cheng et al., 2020b) toward revealing neuronal complications. Werner and co-workers have indicated additional symptoms of several cases, such as acute necrotizing encephalopathy, neck stiffness, bilateral ankle clonus, positive Brudzinski, left Babinski, and right Chaddock signs (Werner et al., 2020). Other neuronal symptoms of COVID-19 are ataxia, refractory status epilepticus (Xu et al., 2005), neuron denaturation/necrosis, broad gliocytes hyperplasia with gliosome formation (Yassin et al., 2020), myalgia, dyspnea (Prakash et al., 2020), taste and smell dysfunctions, acute cerebrovascular and oculomotor nerve palsy (Nepal et al., 2020). Mao *et al*. indicated that elevated creatine phosphokinase (CPK), C-reactive protein (CRP), D-dimer, necrotizing myopathy, thick filament myopathy, critical illness myopathy (nonspecific), and acute quadriplegic myopathy are other neural manifestation of COVID-19 (Mao et al., 2020; Suri et al., 2020; Warner, 2020). Reports have also shown other neurological manifestations such as Bickerstaff’s encephalitis, critical illness myopathy, severe lymphopenia, thrombocytopenia and uremia, facial diplegia, and toxin associated myopathy and neuropathy (Wu et al., 2017; Gulati et al., 2020; Zheng et al., 2020). Of the clinical behavioral signs, headache, syncope, agitation, delirium, dysgeusia, fatigue, dizziness, acute confusion, sleep disorders, changed the level of consciousness, and altered mental status, have been observed in COVID-19 patients (Stewart et al., 1992; Dessau et al., 2001; Lau et al., 2004; Wang et al., 2020a; Wang et al., 2020c; Wu et al., 2020c; Dorche et al., 2020; Helms et al., 2020; Mochan and Modi, 2020; Nalleballe et al., 2020). The aforementioned neurological signs are being manifested in 84% of patients with COVID-19 (Wang et al., 2020c; Helms et al., 2020).

Severe respiratory syndrome as one of the critical impairment of COVID-19 may result in systemic hypoxia, hypercarbia, and anaerobic metabolism resulting in neuronal swelling and brain edema/damage (Suri et al., 2020). SARS-CoV-2 also invades to the spinal cord and causes acute inflammation of gray and white matter in the spinal cord (myelitis), which was recognized by the acute flaccid myelitis of lower limbs, urinary and bowel incontinence (Zhao et al., 2020). Evidence has shown a close relationship between COVID-19 and Parkinson disease, increased motor symptoms (e.g., tremor), freezing of gait or dyskinesias, and declined the efficacy of dopaminergic medication (Macht et al., 2007; Zach et al., 2017; Ehgoetz Martens et al., 2018). Interestingly, it seems to be a near linkage between dopamine synthesis pathway and COVID-19 pathophysiology. In this line, dopa decarboxylase, as a regulatory enzyme in dopamine pathway is meaningfully co-expressed with ACE2 receptor. On the other hand, SARS-COV virus downregulates ACE2 in consistent with dopamine synthesis alteration (Kuba et al., 2005). Besides, as dopamine is expressed in the alveolar epithelial cells, it also contributes in lung immunity, as well as what ACE2 does (Bone et al., 2017). Accordingly, considering the critical role of dopamine deficiency in Parkinson’s disease, the SARS-CoV-2 virus may cause such sporadic signs COVID-19 patients (Rietdijk et al., 2017).

Additionally, evidence indicated that CoVs may play an essential function in the pathogenesis of multiple sclerosis (Saleki et al., 2020). The CoVs isolated from multiple sclerosis patients were neutralized using the patients’ serum. This revealed the destructive role of CoVs in the pathogenesis of multiple sclerosis (Burks et al., 1980). Growing studies are evaluating the use of immunomodulatory/disease-modifying agents in multiple sclerosis patients with COVID-19. Results declared an increased risk of COVID-19 complications in those treated patients (Baysal-Kirac and Uysal, 2020; Ramanathan et al., 2020). Decision on continuing/stopping the immunotherapy in these patients is closely dependent on disease severity and activity (Giovannoni et al., 2020).

Orsucci and co-others have revealed that there are olfactory and gustatory function impairments as common neural disorders in patients of COVID-19 (Orsucci et al., 2020). It has shown in CNS-CoV disease, there is a lower level of lymphocytes, eosinophils and a higher level of neutrophils as well as monocyte (Saleki et al., 2020). Also, Toscano *et al.* observed Guillain–Barré syndrome, lower limb weakness and paresthesia, facial diplegia followed by ataxia and paresthesia, flaccid tetraparesis or tetraplegia in COVID-19 (Toscano et al., 2020). Researchers in several cases observed tonic-clonic seizure, anxiety, psychotic symptoms, meningeal irritation signs, extensor plantar response, encephalitis, dysphagia, dysarthria, bulbar impairment and massive hemorrhagic conversion (Wang et al., 2020a).

## THE Pathophysiological Mechanistic Pathways of Coronaviruses in Central Nervous System

Experimental evidence has indicated that coronaviruses invade to neurons and glial cells to induce an unfolded protein response (UPR) regarding necroptosis in neuronal cells (Meessen-Pinard et al., 2017). As previously mentioned, coronaviruses caused neuronal damages and death along with related neuroinflammatory responses (Morfopoulou et al., 2016). There are multiple mechanisms by which SARS-CoV-2 enters the CNS and causes associated complications. Those mechanisms are blood-mediated contamination (hematogenous), neuronal-mediated infection (neurogenic), immunodeficient related damage, direct respiratory infection, and hypoxic injury (Ahmed et al., 2020) which are described as following. During the hematogenous manner, CoVs crossed the blood-brain barrier (BBB) and entered the brain. This occurs via two mechanisms, by direct penetration of the virus particle crossing the BBB or by hijacking peripheral blood cells (Bohmwald et al., 2018). In the latter way of invasion, Human coronavirus OC43 (HCoV-OC43) accesses the CNS via the neurogenic way to be appeared in the cell bodies and dendrites of olfactory neurons, then spread in hippocampus, cortex and spinal cord (Niu et al., 2020a). During the viremia phase of illness, BBB disruption causes a direct virus entrance to the brain. Spreading/disseminating of SARS-CoV-2 from the cribriform bone in nearby proximity to the olfactory bulb, and brain causes in seven days (Baig et al., 2020). Besides, peripheral invasion of nerve terminals by CoVs through the connected synapse leads to the virus entry to the CNS (Ahmad and Rathore, 2020). Additionally, systemic hypoxia resulted from severe pneumonia causes vasodilatation, anaerobic metabolism, hypoxia and accumulation of toxic compounds lead to brain damage (Tu et al., 2020).

One of the most widely accepted neuropathological mechanisms of SARS-CoV-2 is hyper-inflammatory state (Yavarpour-Bali and Ghasemi-Kasman, 2020). Accordingly, the immune-mediated damage is resulted from cytokine storms, as well as the activation of T lymphocytes, endothelial cells, and macrophages which leads into vascular leakage, coagulation, and end-organ damage (Mehta et al., 2020; Tveito, 2020). It was shown that coronavirus triggers innate immunity associated with the release of microglial-induced INF-α/β (Savarin et al., 2018). In this regard, several cytokines and chemokines are released by microglia and astrocytes such as IL-1α, IL-1β, IL-6, IFN-γ, TNF-α, and CXCL10 (Joseph et al., 1993). Li *et al.* indicated the increased levels of many inflammatory mediators in the cerebrospinal fluid, including IL-6, IL-8, MCP-1, and granulocyte-macrophage colony-stimulating factor (GM-CSF) in COVID-19 patients (Li et al., 2016). During early stage of CoV neuroinfection, CXCL10 and CXCL9 are present in the peripheral blood of patient affected by IFN-γ (Jiang et al., 2005). Experiment has shown that CoVs play a destructive role in acute disseminated encephalomyelitis (ADEM) correlated with increased inflammatory mediators such as IL-6, IFN-γ, TNF-α, CXCL9, and CXCL10 (Kothur et al., 2016). It has been demonstrated that there is a direct correlation between the levels of IL-1β, IL-6, IL-8, TNF-α, IL-10 and COVID-19 central inflammatory complications such as neuromyelitis optica (also known as Devic's disease), transverse myelitis, acute disseminated encephalomyelitis, amyotrophic lateral sclerosis, herpes simplex encephalitis, Parkinson’s disease, traumatic brain injury, epilepsy, and stroke (Vezzani et al., 2002; Rodney et al., 2018; Vezzani et al., 2019; West et al., 2019). In this line, it has shown that IL-2 and IL-2 receptors (IL-2R) have important signals for T cell activation via Janus kinase/signal transducer and activator of transcription (JAK/STAT) signaling pathway (Fu et al., 2018; Shi et al., 2020). The transcription factor nuclear factor-κB (NF-κB) is another essential regulator in immune system, which is activated in lung inflammatory immunopathology-induced by SARS-CoV (DeDiego et al., 2014; Catanzaro et al., 2020).

As previously mentioned, studies have suggested several mechanisms for entering the SARS-CoV-2 to the nervous system, although the exact mechanism is not clear (Yavarpour-Bali and Ghasemi-Kasman, 2020). Scientists have suggested that coronaviruses enter the olfactory bulb/epithelium, then penetrates to CNS. So, make the anosmia or hyposmia as the neural manifestation of COVID-19. Recently additional studies have suggested different mechanisms for anosmia in COVID-19, such as olfactory cleft syndrome, mucosal obstruction, direct damage of olfactory sensory neurons, impairment of the olfactory perception center and cytokine storm in the brain (Yazdanpanah et al., 2020). Released inflammatory factors altered the penetrance of the BBB and increased inflammatory cascade (Singhi, 2011). Studies have also shown that deficiency in neuronal endoplasmic reticulum (ER) leads to the activation of UPR-induced by SARS-CoV (Chan et al., 2006; Ron and Walter, 2007). Until now, some related signaling pathways have shown functional roles in the UPR processing, such as ATF6, phospho-extracellular signal-regulated kinase (p-ERK)/eIF2-alpha and IRE1/XBP1 (Ron and Walter, 2007). Favreau *et al.* indicated that HCoV-OC43 induced UPR and causes neuronal death by caspase-3 activation and nuclear fragmentation (Favreau et al., 2009). From another mechanistic point, studies suggested that SARS-CoV-2 induces severe inflammation that leads to thrombosis. SARS-CoV-2 also binds to toll-like receptors (TLR) and causes the synthesis and liberation of IL-1. As a matter of fact, TLRs activate biochemical cascade by inflammasome activation as well as type I interferon (IFN) which is released as an important player against viral infection (Marchetti et al., 2018; Conti et al., 2020; Vaninov, 2020).

## Role of RENIN-ANGIOTENSIN System in the Neuronal Manifestations of Coronaviruses

It has been shown that SARS-CoV-2 mainly enters the CNS via the ACE2 or TMPRSS2 receptors. These receptors are expressed in the glial cells of brain/spinal cord and thereby facilitates the invasion of coronavirus to the spinal cord, which is essential for the host cell entry of SARS-CoV-2 and also plasma membrane fusion (El Tabaa and El Tabaa, 2020; Nemoto et al., 2020). Also, it has been indicated that when coronavirus enters the cells, ACE2 will break and shed by ADAM Metallopeptidase Domain 17 (ADAM17) into the membrane space (Li and De Clercq, 2020). Studies suggested that phosphorylation of ACE2 at Ser680 inhibits ubiquitination of ACE2 and also increase related membrane expression (Amraei and Rahimi, 2020). It has been indicated that renin-angiotensin system (RAS), including angiotensin II (Ang II), ACE, ACE2, angiotensin type-1 receptor (AT1R), angiotensin type-2 receptor (AT2R), and Mas receptor (MAS), plays critical physiological functions. Research suggested that Ang II prevents COVID-19 infection through binding to ATR1 and activating ACE2 internalization, then declining ERK1/2 and p38 mitogen-activated protein kinase (MAPK) pathway (Koka et al., 2008; Fernandes et al., 2011; Divani et al., 2020). Recent reports indicated that Ang II act via two G protein-coupled receptors (GPCR) such as AT1R, angiotensin type-2 receptor (AT2R) which expressed in human lung tissue. Besides, the activations of Ang II can be mediated by AT1R through enhancing several signaling pathways such as MAPK/ERK, IP3/diacylglycerol, tyrosine kinases, and NF-κB (Balakumar and Jagadeesh, 2014; El Tabaa and El Tabaa, 2020). In a parallel way, AT1 stimulates monocytes, macrophages and vascular smooth muscle cells to generate TNF-α and IL-6 (Balakumar and Jagadeesh, 2014). Additionally, Ang II promotes vasoconstriction, released pro-inflammatory cytokine, vascular endothelial dysfunction, and platelet aggregation (Nakashima et al., 2006; Shatanawi et al., 2011). There is also a relationship between Ang II and endothelin-1 (ET-1). Indeed ET-1 has an important function in Ang II-induced endothelial dysfunction and platelet activation through inducing IL-6 release (Touyz and Schiffrin, 1993; Browatzki et al., 2000). In order to reduce SARS-CoV-2 entry and related side effects, ACE2 activity should be declined. It has been found that ACE2 is a critical enzyme in the RAS, which has a critical function role in the human body. In this pathway, renin generated in the kidneys cleaves angiotensinogen from the liver, producing Ang I and then is cleaved by ACE into Ang-II (the substrate of ACE2). Ang I binds to the AT1R and AT2R as well as the RAS system has an important function in SARS-CoV-2 infection (Battagello et al., 2020).

In addition to the critical role of blood, hypoxia, ACE2, neuroinflammation in the neuronal pathogenesis of COVID-19, modulating RdRP/3-chymotrypsin-like protease (3CLpro) and papain-like protease (PLpro), as critical enzymes involved in the replication of SARS-CoV-2, is of great importance. There are also several receptors, namely CD209L (L-SIGN), CD209 (DC-SIGN), neuropilin receptors (NRPs), and CD147/Basigin, which facilitate SARS-CoV-2 entry (Amraei and Rahimi, 2020). As described, there is a close interconnection between the aforementioned dysregulated signaling pathways. In this line, providing multi-target agents capable of a simultaneous modulation of the aforementioned targets could pave the road against COVID-19 neurological manifestations.

## Importance of Natural Products in Combating COVID-19 General Manifestations

The widespread pandemic of COVID-19 disease, by infecting millions of people, and thousands of killing around the world, has triggered researchers to make a diligent effort regarding finding potential drugs or vaccines against SARS-CoV-2. However, these efforts have not yet reached credible drugs due to the inherent complexity of the SARS-CoV-2 pathogenicity/complications (Sharma et al., 2020). Due to their simultaneous effects on multi-therapeutic targets and low side effects, phytochemicals including alkaloids, flavonoids, polyphenols, quinones, and terpenoids are of the most promising options for finding effective treatment against SARS-CoV-2 (Efferth and Koch, 2011; Mani et al., 2020).

Recent studies showed that three main targets, including main proteases, as well as S protein interaction with ACE2, have attracted the most attention of researchers to discover effective drugs against SARS-CoV-2 from phytochemicals. Additionally, phytochemicals potentially target neuroinflammation to combat related neuronal signs in COVID-19.

## Potential of Phytochemicals Against COVID-19 Neurological Associations

Recently, no drug or vaccine has been developed for the treatment/prevention of SARS-CoV-2. Phytochemicals have shown to play critical antiviral biological activities and health benefits in CNS (Kähkönen et al., 1999). As previously mentioned, there are several major targets for phytochemicals against coronavirus such as ACE2, spike protein, TMPRSS2, 3CLpro, RdRp and PLpro, which among them ACE2 plays an important role regarding the initial stage of SARS-CoV-2 invasion into the cells/neurons (Huang et al., 2020b). Also, 3CLpro and PLpro play vital roles in SARS-CoV-2 maturation and replication (Xue et al., 2008; Ryu et al., 2010a).

The potential of phytochemicals in suppressing neuroinflammation induced by SARS-CoV-2 has also made them promising agents in combating neuronal signs of COVID-19.

### Phytochemicals Inhibit Neuroinflammation and Neural Manifestations in COVID-19

As previously mentioned, hyper-inflammation is one of the critical neuropathological mechanisms of SARS-CoV-2 in line with the release of IL-2, IL-6, IL-7, IL-10 and TNF-α (Yang et al., 2020). Studies also suggested elevated levels of IL-8, MCP-1, IFN-γ, CXCL9, CXCL10 and GM-CSF in COVID-19 patients (Li et al., 2016; Marchetti et al., 2018; Conti et al., 2020; Vaninov, 2020) regarding triggering the neuronal manifestations. Systemic inflammation following the leukocyte activation prior to its BBB migrating is another major mechanism toward viral neurological complications (Campbell et al., 2014). The released inflammatory agents change the BBB permeability, triggers the neuroinflammatory flows and drive neuronal hyper-excitability through the activation of glutamate receptors, leading to acute seizure (Libbey et al., 2011; Yavarpour-Bali and Ghasemi-Kasman, 2020). Considering the crucial role of inflammation in the neuropathogenesis of COVID-19, phytochemicals with neuronal anti-inflammatory effects could pave the road in combating related neuronal manifestations. Recent reports also have declared the critical role of phytochemicals in health care through their antiviral (Fitriani et al., 2020) and the inhibition of neuroinflammatory-interconnected pathways (Abbaszadeh et al., 2020). Phytochemicals with potential antioxidant and anti-inflammatory effects (e.g., carotenoids and polyphenols) interact with major transcription factors such as Nrf2 and NF-κB (Iddir et al., 2020).

**FIGURE 1:**
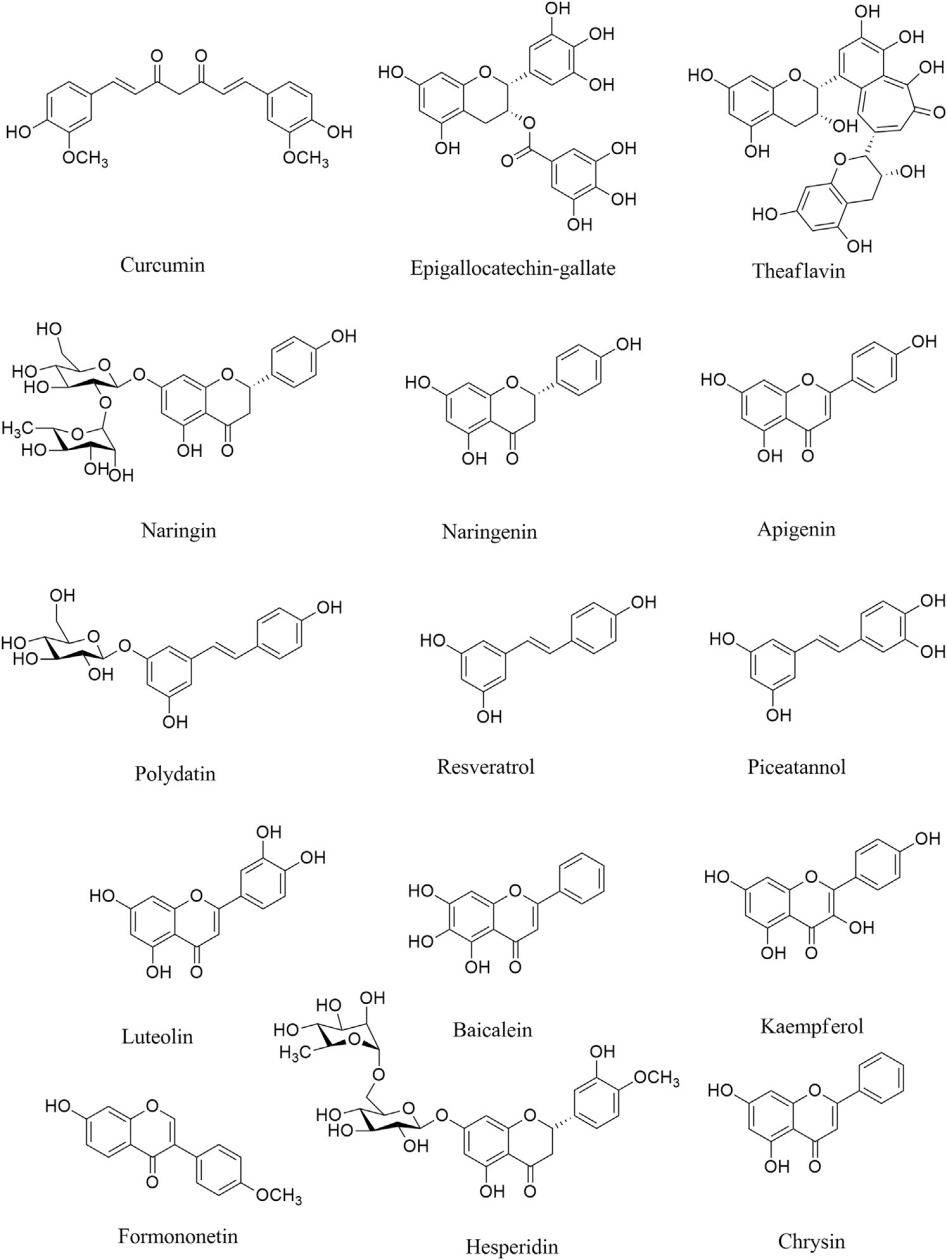
Chemical structures of selected polyphenols/flavonoids with the potential of being used against COVID-19 neurological manifestations.

Naringin (Figure 1) is a phenolic phytochemical belonging to the flavonoid class, possessing anti-neuroinflammatory (Chen et al., 2016; Chtourou et al., 2016; Ngwa et al., 2020), and antiviral (Ng et al., 1996) effects with the potential of being used in the prevention/treatment of COVID-19 (Dabaghian et al., 2020). Naringin also inhibits the expression level of cyclooxygenase (COX)-2, inducible nitric oxide synthase (iNOS), IL-1β and IL-6 via suppressing high mobility group box 1 (HMGB1) in COVID-19 (Park et al., 2003; Huang et al., 2020a). It also declined the expression level of p38MAPK to inhibit HMGB1 generation of inflammatory mediators and associated lung injury (Gil et al., 2016; Kim et al., 2019b). According to the critical destructive role of inflammatory mediators in the neurological signs of COVID-19, naringin seems to be a hopeful anti-inflammatory/antiviral candidate in combating related neuronal manifestations. As an aglycone form of naringin, naringenin has similarly shown anti-neuroinflammatory (Nouri et al., 2019; Alberca et al., 2020), and antiviral effects with the potential of being used against COVID-19 (Tutunchi et al., 2020). We have previously shown the neuroprotective potential of naringenin through modulation of inflammatory mediators (NF-κB, TNF-α, IL-1β, etc) and microglia activation in the CNS (Nouri et al., 2019), thereby it could mitigate the neuronal signs of COVID-19 mediated by the inflammatory mediators. As another phenolic compound, resveratrol has shown promising beneficial effects against COVID-19, through the activation of ERK1/2 and SIR1 signaling pathways related to survival, DNA protection (Levy et al., 2020; Ma and Li, 2020), and anti-neuroinflammatory responses (Bastianetto et al., 2015). It also inhibits neuroapoptosis by reducing FGF-2 and suppressing NF-κB (Xu et al., 2018). Considering the critical role of the aforementioned inflammatory mediators in COVID-19 (Yarmohammadi et al., 2020), resveratrol could potentially decline neuroinflammatory signs of COVID-19 patients (Chen et al., 2005). As a major natural derivative of resveratrol, polydatin potentially decline the neural levels of NF-κB, TNF-α, IL-1β, IL-6, IL-8, prostaglandin E2 (PGE2), NO, COX-2, iNOS, matrix metalloproteinase (MMP)-3 and MMP-9, thereby could be a novel agents in combating neuronal inflammatory manifestation in COVID-19 (Lo Muzio et al., 2020). A recent study by Bonucci *et al.*, has also introduced polydatin as a protective phytochemical against COVID-19 (Bonucci et al., 2020). So, focusing on their ameliorating effects against neuroinflammation, as well as related antiviral properties, resveratrol and plydatin derivative could be of candidate phytochemicals in combating neuronal signs of COVID-19.

Consistently, evidence has shown that epigallocatechin gallate (EGCG), as a natural polyphenolic compound, plays important functions such as antitumorigenic, anti-inflammatory, antibacterial, antioxidative, and antiproliferative effects (Chacko et al., 2010; Ge et al., 2018; Mhatre et al., 2020). The anti-neuroinflammatory effects through inhibiting microglia activation, and suppressing inflammatory mediators (Abbaszadeh et al., 2020), as well as antiviral effects of EGCG (Steinmann et al., 2013) make it a potential polyphenol for the treatment of neurological symptom in COVID-19. Green tea with the prominent phytochemicals of such polyphenols, including EGCG, epicatechin gallate, epicatechin and catechin plays both the antiviral (Chojnacka et al., 2020), anti-SARS-CoV-2 (Ghosh et al., 2020) and anti-neuroinflammatory activities (Calis et al., 2020), thereby could play promising role in combating COVID-19 neural complications. EGCG has employed several other mechanisms to suppress SARS-CoV-2 in different steps of virus life cycle (Jang et al., 2020).

As another polyphenol, formononetin declined neuroinflammation by decreasing the levels of TNF-α, IL-6, IL-1β, PGE2, iNOS, and COX-2. Evidence indicated that formononetin inhibited neuroinflammation through suppressing NF-κB signaling pathway, thereby could be a novel drug for the neurological manifestation of COVID-19 (El-Bakoush and Olajide, 2018; An et al., 2020). Formononetin was shown to modulate MAPK, ERK, p38, JNK pathway and downstream mediators to play antiviral effects and inhibit infection-induced inflammation (Wang et al., 2015; Lalani and Poh, 2020). Recent reports also have considered the formononetin as one of major plant-derived secondary metabolites with acceptable effectiveness against COVID-19 (Mirzaie et al., 2020). Consistently, theaflavins are other phenolic compounds with antiviral, anti-inflammatory, antioxidative, and antibacterial effects (Higdon and Frei, 2003; Lambert and Yang, 2003). Theaflavins also suppressed the levels of inflammatory mediators such as COX-2, TNF-α, intercellular adhesion molecule 1 (ICAM-1), and NF-κB mRNA (Mhatre et al., 2020). The aforementioned effects of theaflavins, as well as its antiviral potentials (Zu et al., 2012), could introduce it as a useful treatment against the neurological sign of COVID-19, via modulation of neuronal IL-1β, IL-6, TNF-α, IL-10, glial fibrillary acidic protein and Bax. As well as related interaction with ACE2/spike proteins, and main proteases. Based on molecular dynamic analysis Kumar *et al.* indicated that some other phenolic compounds play important roles in the inhibition of SARS-CoV-2 such as rosmarinic acid, ferulic acid, ursonic acid, piperine, gingerol, curcumin, and silymarin (Kumar et al., 2020). Previously the neuroprotective effects of such plant-derived secondary metabolites have been reported through inhibiting the inflammatory-interconnected mediators (Abbaszadeh et al., 2020; Fakhri et al., 2020b). Among the aforementioned phytochemicals, ferulic acid, silymarin and curcumin possess particular anti-neuroinflammatory effects, in addition to related antiviral effects (Dutta et al., 2009; Borah et al., 2013; Ghosh et al., 2017). The anti-neuroinflammatory effects of curcumin is applied through suppressing microglia cells (Ghasemi et al., 2019). Other flavonoids like luteoloside and baicalein also possess potential modulatory effects against neuroinflammation, toward antiviral effects (Nagai et al., 1995; Cao et al., 2016; Li et al., 2019a; Welcome, 2020).

Among other classes of phytochemical compounds, phytosterols also have shown potential anti-inflammatory effects (Dash et al., 2020). Of those compounds, stigmasterol and β-sitosterol reduced the expression of COX-2, TNF-α, iNOS, IL-6, IL-1β, PGE2 and NF-κB (Philip et al., 2018). Consequently, Krupanidhi *et al.* indicated the antiviral effects of stigmasterol and β-sitosterol against the SARS-CoV-2 by computational studies. So, considering the antiviral and anti-inflammatory potential of stigmasterol and β-sitosterol, they could be potential agents in combating COVID-19 neurological signs (Krupanidhi et al., 2020).

Additionally, several lines of evidence indicated that asiaticoside (a saponin), borneol (a terpene), catalpol (an iridoid) as other phytochemicals declined the neuronal levels of TNF-α, IL-6, TLR4, NF-κB, IL-β and IL-8, thus may be hopeful agents against neurological symptoms in COVID-19 (Welcome, 2020). In fact, since inflammation triggers several cascades of CNS pathogenesis in COVID-19, suppressing related mediators could potentially ameliorate related symptoms. Among other phytochemicals, some alkaloids also show promising anti-inflammatory and antiviral effects (Chen et al., 2015; Powers and Setzer, 2016), with the potential of being used against COVID-19 (Bleasel and Peterson, 2020). This effect of alkaloids was also confirmed by a recent *in silico* study by Garg and Roy. In their study, two alkaloids of sophaline D and thalimonine indicated potential antiviral activities by suppressing main viral proteases (Garg and Roy, 2020) and inflammatory pathways (Varadinova et al., 1996; Pour et al., 2019).

Several other phytochemicals play important roles in the inhibition of SARS-CoV-2 such as sarsasapogenin (a steroidal sapogenin), novobiocin (a coumarin), and alpha terpinyl acetate (a terpenoid) (Kumar et al., 2020). Previously the neuroprotective effects of such plant-derived secondary metabolites have been reported through inhibiting the inflammatory-interconnected mediators (Abbaszadeh et al., 2020; Fakhri et al., 2020b).

Cannabinoids also possess critical anti-inflammatory roles in viral diseases (Walter and Stella, 2004; Rizzo et al., 2020). These compounds are major constituents of the cannabis plant. The physiological roles of cannabinoids and cannabis are primarily mediated by the cannabinoid receptors (CB1R and CB2R), endocannabinoids, and related metabolic enzymes which are widely distributed throughout the body, especially CNS. The mediators of cannabinoid receptors are being considered as potential targets for numerous disorders, including those correlated with inflammation and autoimmune dysregulation (Rizzo et al., 2020). Prevailing evidence are indicating the pivotal anti-inflammatory and immunoregulatory effects of cannabis-derived cannabinoids, through suppressing cytokines, inhibition of immune cell migration/proliferation (Almogi-Hazan and Or, 2020). Besides, selective cannabinoid agonists present a novel way regarding the treatment of virus-associated neuroinflammation. Considering their growing global acceptance for medicinal uses (Onaivi et al., 2020), cannabinoids seem to be of potential agents against inflammatory cytokine and related mortality in COVID-19 (Onaivi and Sharma, 2020).

Overall, phytochemicals with the potential of modulating the immune system and attributed neuronal cytokine storm could pave the road in combating COVID-19 neuronal complications.

### Phytochemicals Inhibit ACE2, and Spike Protein Thereby Neural Manifestations in COVID-19

As previously mentioned, SARS-CoV-2 enters the CNS via the ACE2 or TMPRSS2 receptors (El Tabaa and El Tabaa, 2020; Nemoto et al., 2020). In order to decline SARS-CoV-2 entry to neural cells, ACE2 activity should be declined (Battagello et al., 2020). Spike (S) glycoprotein as the main SARS-CoV-2 structural protein with a critical role in binding to the host cell and protecting the virus against some of the host species antibodies, is another target of phytochemicals (Schoeman and Fielding, 2019).

ACE2 is an enzyme found in the outer membrane of the human cell that acts as a binding site for the S protein. Several studies have shown that there is a strong interaction between ACE2 and S protein. So, blocking ACE2 is also another phytochemical strategy to fight SARS-CoV-2 (Li et al., 2005).

Flavonoids reduce the ACE2 expression through activating Nrf2, thereby combat SARS-CoV-2 (Mendonca and Soliman, 2020; Muchtaridi et al., 2020). Based on the molecular docking mutagenesis study and experimental verification results, hesperidin, chrysin and emodin can be used for COVID-19 treatment (Basu et al., 2020). An *in silico* study indicated that kaempferol, quercetin, and fisetin bind to the hACE2-S-protein complex, near the interface of hACE2 and S protein binding (Pandey et al., 2020). In a recent study by Rebas et al., 2020 the neuroprotective effects of the aforementioned compounds have been shown. So, kaempferol, quercetin and fisetin are of promising flavonoids against COVID-19 neurological signs. Two *in silico* studies showed that quercetin, quercetin 3-glucuronide-7-glucoside, quercetin 3-vicianoside, absinthin, glabridin, and gallic acid gave better binding energy (BE) with ACE2 (Joshi et al., 2020) toward inhibiting COVID-19 (Joshi et al., 2020).

Through the same molecular docking analysis piceatannol also has shown neuroprotective responses (Zhang et al., 2018; Talebi et al., 2020) with the potential of binding to ACE2, thereby playing a critical role in the prevention and treatment of COVID-19 (Wahedi et al., 2020; Ahmad et al., 2020). The phytochemicals, baicalin, scutellarin, and hesperetin, also bind to ACE2, regarding reducing neurological symptoms in COVID-19 (Cheng et al., 2020a). Several *in silico* studies showed that the binding energy of hesperidin with the SARS-CoV-2 spike protein, and main proteases are lower than that of ritonavir, lopinavir, and indinavir. It could introduce hesperidin as an effective antiviral agent. Hesperidin also has shown to counteract the cell damaging induced by virus infection, inflammation and free radicals (Bellavite and Donzelli, 2020). Many of other phenolic compounds, including naringenin, hesperetin, hesperidin, and baicalin, showed potential inhibitory effects on ACE2 activity, thereby showed potential effects on COVID-19 and related neural manifestations (Muchtaridi et al., 2020). In another study, stilbene-based compounds especially resveratrol, are promising candidate phytochemicals acting via disrupting spike protein and human ACE2 receptor complex (Wahedi et al., 2020).

EGCG and theaflavin gallate seem to be of promising phytochemicals in targeting spike-protein central channel of SARS-CoV-2 (Maiti and Banerjee, 2020). In a recent study by Kulkarni et al., 2020 some terpenoids such as carvacrol, geraniol, anethole, l-4-terpineol,cinnamyl acetate, thymol and pulegone, and other phenolic as cinnamaldehyde were effective antiviral agents with potential inhibitory effects on viral spike protein. In this line, nimbin (a triterpenoid) and curcumin (polyphenol) showed high binding affinity regarding interacting with ACE2 and the S protein (Maurya et al., 2020). Consistently, Chen and Due estimated the BE of ACE2 interaction with the flavonoid glycoside scutellarin and the triterpenoid glycyrrhizin as a -14.9 and -9 kcal/mol, respectively, that were more strong than other studied phytochemicals including baicalin, hesperetin, and nicotianamine (Chen and Du, 2020). A study by Vardhan *et al.*, showed that one hundred fifty-four analogous of limonoids and triterpenoids showed potential inhibitory effects on ACE2, 3CLpro, PLpro, spike protein, and RdRp. Another *in silico* study also showed that limonin, obacunone, ursolic acid, glycyrrhizic acid, 7-deacetyl-7-benzoylgedunin, maslinic acid, and corosolic acid effectively target SARS-CoV-2 proteins (Vardhan and Sahoo, 2020).

**FIGURE 2:**
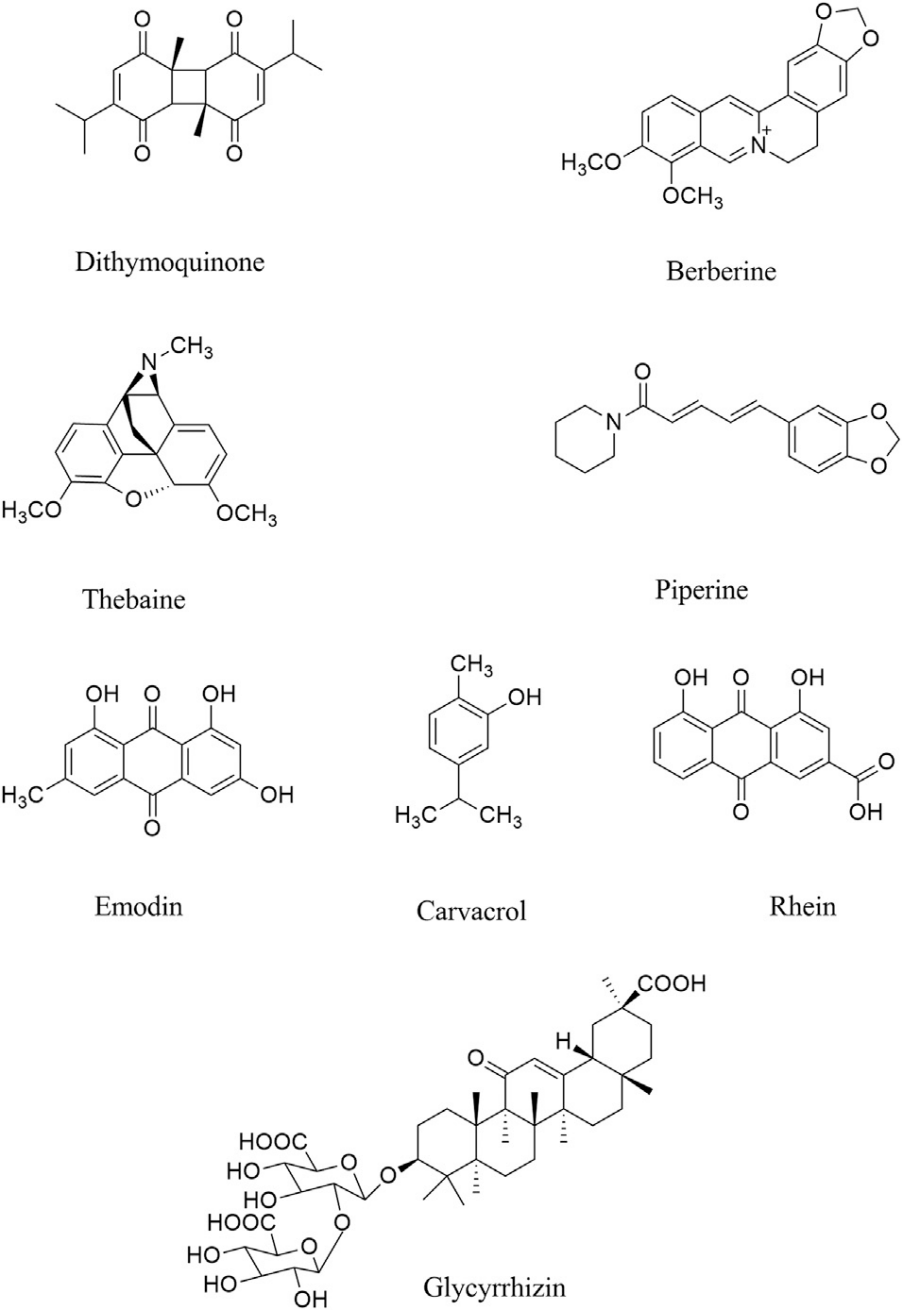
Chemical structures of selected alkaloids/terpenes/quinones with the potential of being used against COVID-19 neurological manifestations.

Evaluated by molecular docking analysis, dithymoquinone (a quinone, Figure 2) showed neuroprotective responses (Zhang et al., 2018; Talebi et al., 2020) through binding to ACE2, to show key roles in the prevention and treatment of COVID-19 (Wahedi et al., 2020; Ahmad et al., 2020). As a potential phytochemical of *Nigella sativa L.* (Ranunculaceae), dithymoquinone, with binding affinity of -8.6 kcal/mol, showed a higher potential of binding at SARS-CoV-2 ACE2 (Ahmad et al., 2020). According to the molecular modeling results on SARS-CoV-2, a new indazole alkaloid from the seeds of *N. sativa*, nigellidine meaningfully bind to active sites of SARS-CoV-2 (Maiti et al., 2020).

Parvez and co-workers, in an *in silico* study, showed that two chalcones azobechalcone (binding energy [BE], −14.4 kcal/mol) and isolophirachalcone (BE, −12.8 kcal/mol) as well as two alkaloids fangchinoline (BE, −12.5 kcal/mol) and tetrandrine (BE, −12.6 kcal/mol) have shown high binding affinity to S protein of SARS-CoV-2 (Parvez et al., 2020). Also, three alkaloids, including cepharanthine, fangchinoline, and tetrandrine inhibited the S protein of Human-CoV-OC43 expression at 5 µM (Kim et al., 2019a), as previously showed anti-inflammatory roles in viral diseases. In another study, Ho and co-workers showed that anthraquinone emodin (IC_50_, 200 µM) blocked the interaction between ACE2 and S protein (Okamoto et al., 2001; Ho et al., 2007).

In a survey by Niu *et al.* glabridin, genistein, chrysoeriol, and tectorigenin have been introduced as phytochemicals affecting miRNAs of ACE2 (Niu et al., 2020b). *In vitro* investigation showed that rhoifolin, δ-viniferin, myritilin, homoflavone A, lactucopicrin15-oxalate, nympholide A, afzelin, biorobin, phyllaemblicin B, cyanidin, baicalin, scutellarin, glycyrrhizin, tangeretin, pro-cyanidin, nobiletin, brazilein, galangin, acetoxychavicol acetate (ACA) and delphinidin are among other phytochemicals which inhibit ACE to suppress COVID-19 (Maroli et al., 2020; Muchtaridi et al., 2020).

Recent reports confirmed that there are several other phytochemicals, which inhibited ACE2 activity, including neohesperidin, nobiletin, scutellarin, nicotinamin, and glycyrinodin (Muchtaridi et al., 2020). As another natural product with antiviral properties, glycyrrhizic acid binds to ACE2, thereby could be used for treatment of COVID-19 neurological signs (Pilcher, 2003). Luteolin also inhibited furin proteins which breakdown the S protein in SARS-CoV. Similarly, herbacetin inhibited the interaction between S protein and ACE2. Accordingly, these phytochemicals can be useful for treating/managing neurological manifestation of COVID-19 by targeting the ACE2/spike proteins to suppress the penetration/attachment of SARS-CoV-2 to the CNS cells, what triggers the neurological signs (Wu et al., 2020b). Overall, evidence has shown berberine, thebaine, piperine (as alkaloids), withaferin A (steroidal lactone), nimbin, embelin, cafestol, murrayanine, murrayaquinone-A and andrographolide are phytochemicals with the potential antiviral effects for example through binding to spike protein in SARS-CoV-2, as well as ACE2 receptor (Grover et al., 2011; Boukhatem and Setzer, 2020; Gupta et al., 2020; Parida et al., 2020). Consistent docking results showed the same acceptable inhibitory effects against SARS-CoV-2.

The main phytochemicals with reported inhibitory effects on ACE2 and spike proteins are presented in Table 1.

**TABLE 1:** Selected/candidate phytochemicals with inhibitory effects on ACE2, spike proteins, protease, and RdRP in combating COVID-19 neurological signs.

Phytochemical class	Compound	Study type	References
		ACE2 interaction
Alkaloid	Nicotianamine	*In silico*	(Chen and Du, 2020)
Flavonoid	Baicalin	*In silico*	(Cheng et al., 2020a; Chen and Du, 2020)
Flavonoid	Chrysin	*In silico*	(Basu et al., 2020)
Flavonoid	Fisetin	*In silico*	(Pandey et al., 2020)
Flavonoid	Hesperetin	*In silico*	(Chen and Du, 2020)
Flavonoid	Kaempferol	*In silico*	(Pandey et al., 2020)
Flavonoid	Naringenin	*In silico*	(Muchtaridi et al., 2020)
Flavonoid	Quercetin	*In silico*	(Joshi et al., 2020; Williamson and Kerimi, 2020)
Flavonoid	Scutellarin	*In silico*	(Chen and Du, 2020)
Polyphenol	Curcumin	*In silico*	(Maurya et al., 2020)
Polyphenol	Piceatannol	*In silico*	(Wahedi et al., 2020)
Polyphenol	Resveratrol	*In silico*	(Wahedi et al., 2020)
Quinone	Dithymoquinone	*In silico*	(Ahmad et al., 2020)
Terpenoid	Glycyrrhizin	*In silico*	(Chen and Du, 2020)
Terpenoid	Nimbin	*In silico*	(Maurya et al., 2020)
**Spike protein interaction**			
Alkaloid	Berberine	*In silico*	(Maurya et al., 2020)
Alkaloid	Cepharanthine	*In vitro*	(Kim et al., 2019a)
Alkaloid	Piperine	*In silico*	(Rout et al., 2020)
Alkaloid	Thebaine	*In silico*	(Maurya et al., 2020)
Alkaloid	Fangchinoline	*In silico, In vitro*	(Kim et al., 2019a; Parvez et al., 2020)
Alkaloid	Tetrandrine	*In silico, In vitro*	(Kim et al., 2019a; Parvez et al., 2020)
Flavonoid	Epigallocatechin gallate	*In silico*	(Maiti and Banerjee, 2020)
Flavonoid	Fisetin	*In silico*	(Pandey et al., 2020)
Flavonoid	Isolophirachalcone A	*In silico*	(Parvez et al., 2020)
Flavonoid	Quercetin	*In silico*	(Pandey et al., 2020)
Flavonoid	Theaflavin	*In silico*	(Maiti and Banerjee, 2020)
Phenolic	Cinnamaldehyde	*In silico*	(Kulkarni et al., 2020)
Polyphenol	Curcumin	*In silico*	(Maurya et al., 2020)
Polyphenol	Resveratrol	*In silico*	(Wahedi et al., 2020)
Quinone	Emodin	*In vitro*	(Okamoto et al., 2001; Ho et al., 2007; Ho et al., 2007)
Terpenoid	Carvacrol	*In silico*	(Kulkarni et al., 2020)
Terpenoid	Glycyrrhizin	*In silico*	(Chen and Du, 2020)
Terpenoid	Nimbin	*In silico*	(Maurya et al., 2020)
Terpenoid	Saikosaponin	*In silico*	(Sinha et al., 2020)
**RdRP blockers**			
Alkaloid	6-Acetonyldihydrochelerythrine	*In silico*	(Pandeya et al., 2020)
Alkaloid	Allocryptopine	*In silico*	(Pandeya et al., 2020)
Alkaloid	Cepharanthine	*In silico*	(Ruan et al., 2020)
Alkaloid	Fangchinoline	*In silico*	(Parvez et al., 2020)
Alkaloid	Protopine	*In silico*	(Pandeya et al., 2020)
Alkaloid	Tetrandrine	*In silico*	(Parvez et al., 2020)
Flavonoid	Apigenin	*In silico*	(Rameshkumar et al., 2020)
Flavonoid	Cyanidin	*In silico*	(Rameshkumar et al., 2020)
Flavonoid	Delphinidin	*In silico*	(Rameshkumar et al., 2020)
Flavonoid	Hesperidin	*In silico*	(Singh et al., 2020)
Flavonoid	Isolophirachalcone A	*In silico*	(Parvez et al., 2020)
Flavonoid	Myricetin	*In silico*	(Singh et al., 2020)
Flavonoid	Theaflavin	*In silico*	(Lung et al., 2020; Singh et al., 2020)
Polyphenol	Epigallocatechin gallate	*In silico*	(Singh et al., 2020)
Polyphenol	Gallic acid	*In silico*	(Abd El-Aziz et al., 2020)
Polyphenol	Resveratrol	*In silico*	(Abd El-Aziz et al., 2020)
**Main protease inhibitors**			
Alkaloid	Berberine	*In silico*	(Narkhede et al., 2020)
Alkaloid	Fangchinoline	*In silico*	(Parvez et al., 2020)
Alkaloid	Solanine	*In silico*	(Hasan et al., 2020)
Alkaloid	Triptanthrine	*In silico*	(Narkhede et al., 2020)
Flavonoid	Amentoflavone	*In vitro*	(Ryu et al., 2010a)
Flavonoid	Apigenin	*In vitro*	(Ryu et al., 2010a)
Flavonoid	Fortunellin	*In silico*	(Panagiotopoulos et al., 2020)
Flavonoid	Hesperidin	*In silico*	(Adem et al., 2020a)
Flavonoid	Isolophirachalcone	*In silico*	(Parvez et al., 2020)
Flavonoid	Luteolin	*In vitro*	(Ryu et al., 2010a)
Flavonoid	Narcissin	*In silico*	(Owis et al., 2020)
Flavonoid	Naringenin	*In silico*	(Kim et al., 2019b)
Flavonoid	Oolonghomobisflavan-A	*In silico*	(Bhardwaj et al., 2020)
Flavonoid	Papyriflavonol	*In vitro*	(Park et al., 2017)
Flavonoid	Quercetin	*In vitro*	(Ryu et al., 2010a; Nguyen et al., 2012)
Flavonoid	Rutin	*In silico*	(Adem et al., 2020a)
Iridoid	Geniposide	*In silico*	(Rahman et al., 2020)
Lignan	Savinin	*In vitro*	(Wen et al., 2007)
Polyphenol	Dieckol	*In vitro*	(Park et al., 2013)
Polyphenol	Gallocatechin-3-gallate	*In silico*	(Ghosh et al., 2020)
Quinone	Rhein	*In silico*	(Narkhede et al., 2020)
Quinone	Tanshinone I	*In vitro*	(Park et al., 2012)
Terpenoid	1,8-cineole	*In silico*	(Sharma and Kaur, 2020)
Terpenoid	Andrographolide	*In silico*	(Enmozhi et al., 2020)
Terpenoid	Betulinic acid	*In vitro*	(Wen et al., 2007)

### Phytochemicals Inhibit RdRp, 3CLpro and PLpro, Thereby Neural Manifestations in COVID-19

Ongoing studies are consisting on the key role of RdRp, 3CLpro and PLpro, in the neuropathogenesis of SARS-CoV-2*.* Proteases especially 3CLpro and PLpro, play critical roles in SARS-CoV-2 maturation and replication, and are of the main targets of anti-SARS-CoV-2 phytochemicals (Xue et al., 2008; Ryu et al., 2010a; Shamsi et al., 2016). Polyphenols, especially flavonoids, are among the phytochemicals with anti-SARS effects through inhibiting proteases (Senthilvel et al., 2013; Shamsi et al., 2016; Annunziata et al., 2020). Adem and co-workers, in a molecular docking study on 80 flavonoids showed that 24 of them had suitable interaction with the main protease of SARS-CoV-2, of which hesperidin and rutin had the highest interaction (Adem et al., 2020a). In another *in silico* report, four hundred fifty-eight flavonoids were screened, which among them apigenin 7-(6″-malonylglucoside), cyanidin-3-(*p*-coumaroyl)-rutinoside-5-glucoside, delphinidin 3-*O*-beta-D-glucoside 5-*O*-(6-coumaroyl-beta-D-glucoside), albireodelphin, and (-)-Maackiain-3-*O*-glucosyl-6″-*O*-malonate possessed the most potential in inhibiting SARS-CoV-2. The aforementioned flavonoids showed the highest binding energy values against RdRP, and S proteins of SARS-CoV-2 (Rameshkumar et al., 2020). Another study on twenty-three flavonoids and twenty-five chalcones compounds, showed that the compounds were capable of blocking main proteases. In their study, cyanidin inhibited RNA polymerase and, quercetin blocked the viral spike. As previously mentioned, RdRp catalyzes SARS-CoV-2 RNA replication and thereby is considered an important target for antiviral drug design. Molecular docking investigation revealed that EGCG, theaflavin, theaflavin-3′-*O*-gallate, theaflavin-3′-gallate, theaflavin 3,3′-digallate, hesperidin, quercetagetin, and myricetin bind to the active site of RdRp (Singh et al., 2020). Overall, flavonoids and indole chalcones could combat SARS-CoV-2 (Vijayakumar et al., 2020). Additional evidence confirmed that quercetin and kaempferol possess beneficial anti-inflammatory, antioxidant, antiviral, antiallergic effects which potentially inhibits SARS-CoV 3CLpro, PLpro, and S protein (Di Pierro et al., 2020). Accordingly, docking evidence indicated quercetin and kaempferol as promising compounds against SARS-CoV-2. So, these phytochemicals could decline neurological manifestations in COVID-19 patients (Ryu et al., 2010b). In a recent *in silico* report by Gorla et al. (2020) silymarin, and biochanin A were proposed as bioflavonoids possessing the most acceptable interaction with ACE2/spike protein of SARS-CoV-2. Also, an *in silico* study indicated that naringenin inhibited 3CLpro chains, thereby may be a promising phytochemical for alleviating neurological symptoms in COVID-19 patients (Kim et al., 2019b). Papyriflavonol A as a prenylated flavone inhibited the PLpro and 3CLpro of SARS-CoV at 3.7 and 103.6 µM, respectively (Park et al., 2017). Also, Ryu and co-workers showed that a biflavonoid, amentoflavone, blocked the 3CLpro at 8.3 µM while apigenin, luteolin, and quercetin inhibited the enzyme at 280.8, 20.2, and 23.8 µM, respectively (Ryu et al., 2010a; Yao et al., 2018; Istifli et al., 2020). Oolonghomobisflavan-A (Bhardwaj et al., 2020), narcissin (Owis et al., 2020), isolophirachalcone (Parvez et al., 2020), fortunellin (Panagiotopoulos et al., 2020), dieckol (Park et al., 2013), gallocatechin-3-gallate (Ghosh et al., 2020) are other polyphenols with inhibitory effects on SARS-CoV-2 proteases.

Theaflavins, a group of polyphenols formed after the fermentation of green tea, have a very strong affinity to bind to RdRp (Lung et al., 2020; Singh et al., 2020). Lung and co-workers reported that theaflavin had a high affinity for RdRp of SARS-CoV2, SARS-CoV, and MERS-CoV (Lung et al., 2020). Also, Singh et al. (2020) showed that theaflavin-3,3′-digallate, theaflavin-3′-gallate, theaflavin-3′-*O*-gallate, and theaflavin had the highest affinity for RdRp with -9.9, -9.6, -9.6, and -9.3 kcal/mol bonding energy, respectively. EGCG and hesperidin (Singh et al., 2020), isolophirachalcone A (Parvez et al., 2020), gallic acid and resveratrol (Abd El-Aziz et al., 2020) are other polyphenols with anti-SARS-CoV-2 activities through the high binding affinity to RdRp.

Of other classes of phytochemicals, solanine is a steroidal alkaloid that interacts with two clusters of amino acids of the C3-like protease. The first cluster consists His163, His164, Met165, and Pro168 and the latter contains Asp187, Gln189, and Ala191 (Hasan et al., 2020). There are several other alkaloid that interact with C3-like protease such as solasurine, omatidenol, cycloartanol, diosgenin, lupeol and purpurin (Hasan et al., 2020). Besides, the alkaloids including cepharanthine (Ruan et al., 2020), fangchinoline and tetrandrine (Parvez et al., 2020), protopine, 6-Acetonyldihydrochelerythrine, and allocryptopine (Pandeya et al., 2020) showed strong binding to SARS-CoV-2 RdRp in docking studies.

Nsp15 is responsible for protein interference with the innate immune response, which is essential in the function of coronavirus. Studies indicated that sarsasapogenin, ursonic acid, apigenin, curcumin, ajmalicine, novobiocin, silymarin, alpha amyrin, pomolic acid, carnosol, asiatic acid, reserpine, betulinic acid, platanic acid, taspine, alphitolic acid, taxifolin, wogonin, chlorogenic acid, afromosin, gliotoxin, psoralen, carinatine rhinacanthin, caffeic acid, coriandrin, scopoletin, cordycepin, ricinoleic acid, alpha asarone, allicin and aranotin as other phytochemicals, can bind to Nsp15 protein, thereby could be useful factors for inhibitors of COVID-19 (Kumar et al., 2020; Umesh et al., 2020). In a research by Adem *et al.*, showed the beneficial effects of caffeic acid derivatives were shown as inhibitors of SARS-CoV-2, via inhibition of COVID-19 Nsp15, main proteases, and spike protein (Adem et al., 2020b).

In addition to alkaloids and flavonoids, terpenoids and quinones are other phytochemicals with inhibitory effects on main proteases of SARS-CoV-2. In an *in silico* study, some natural products against SARS-CoV-2 anthraquinones such as rhein and crysophanic acid as well as the alkaloids such as indican, indigo, berberine, tryptanthrine and terpenes (e.g., bicylogermecrene and glycyrrhizin) showed a strong interaction with SARS-CoV-2 main protease. In their study based on the lowest binding energy, rhein (BE, −8.9 kcal/mol) and tryptanthrine (BE, −8.2 kcal/mol) were introduced as suitable candidates against SARS-CoV-2 (Narkhede et al., 2020). Andrographolide (Enmozhi et al., 2020), 1,8-cineole (Sharma and Kaur, 2020), betulinic acid and savinin (Wen et al., 2007), geniposide (Rahman et al., 2020), and tanshinone I (Park et al., 2012) are other phytochemicals with anti-SARS-CoV-2 activities via the blocking the SARS-CoV-2 proteases. In a similar study, silibinin, dihydrorobinetin, peonidin, robinetin, 5-deoxygalangin, scutellarein, purpurin, isorhamnetin, tricetin, gossypetin, norathyriol, coumestrol, isosakuranetin, pectolinarigenin, tangeritin, nobiletin, pratensein, hispidulin, baicalein, morin, urolithin A, acacetin, pelargonidin, irilone, pinocembrin, malvidin, dalbergin, butein, biochanin A, fustin, 5-hydroxyflavone, pinostrobin, pinobanksin, datiscetin, galangin, cyanidin, daidzein, glycitein, wogonin, phloretin, urolithin B, angolensin, pinosylvin, formononetin, liquiritigenin, prunetin, alpinetin, biochanin A, rhapontigenin, equol, piceatannol, isorhapontigenin, danshensu, eugenin, sinapic acid, pterostilbene, pyrogallol, resacetophenone, syringic acid, *p*-coumaric acid, paeonol, protocatechuic acid, tyrosol, catechol, 4-ethylphenol and cinnamic acid as natural product binding to SARS-CoV-2 RdRp (Kurokawa et al., 2001; Bosch-Barrera et al., 2020; Singh et al., 2020). Consistently, based on an study of Umesh et al. (2020) carnosol, rosmanol, and arjunglucoside-I, as natural phytochemicals have shown potential inhibitory effects on SARS-CoV main protease using molecular docking approach. In a recent study by Chojnacka *et al.*, some biologically active phytochemicals like quercetin, betulinic acid, luteolin, indigo, aloeemodine, and quinomethyl triterpenoids, or gallates were of potential key antiviral agents in blocking viral proteases (Chojnacka et al., 2020). Additional studies have shown several phytochemicals such as 18-hydroxy-3-epi-alphayohimbine, vincapusine, alloyohimbine, and gummadiol, toward the inhibition of SARS-CoV 3CLpro, SARS-CoV-2 3CLpro, and MERS-CoV 3CLpro toward the treatment of COVID-19 neuronal associations (Bhardwaj et al., 2020). Phytochemicals with the potential of inhibiting RdRP and proteases are also presented in Table 1.

## Pharmacokinetic Interaction and BBB Permeability of Phytochemicals: AN Approach to Novel Delivery Systems

However, the neuroprotective effects of such phytochemicals have been provided in several studies, estimations of the permeability through the BBB of the phytochemicals were assessed by the SwissADME program (Daina et al., 2017). Information on the estimations of permeability through the BBB, as well as predict absorption, distribution, metabolism, and excretion (ADME) parameters, pharmacokinetic properties, druglike nature and medicinal chemistry friendliness are shown in Supplementary Table S1. Among the fifty-five phytochemicals, the screening of BBB permeability gave fifteen compounds with a positive effect. Among these are the monoterpenoids 1,8-cineole and carvacrol; the alkaloids 6-acetonyldihydrochelerythrine, allocryptopine, berberine, piperine, protopine, thebaine, and triptanthin; the flavonoid chrysin; the quinones dithymoquinone and tanshinone I; the phenolic compounds resveratrol and cinnamaldehyde; and the lignan savinin. To overcome the aforementioned pharmacokinetic drawbacks of some phytochemicals, novel delivery systems are being applied regarding increasing their penetration to BBB. Accordingly, nano-formulations, polymeric micelles, nano-/micro-emulsions, nano-gels, solid lipid nano-particles, polymer composites, and liposome/phospholipid have been studied so far (Abbaszadeh et al., 2020; Fakhri et al., 2020a).

As previously mentioned, inflammatory conditions play critical roles during the pathogenesis of COVID-19 disease. It is worth noting that inflammation could increase the BBB penetration of phytochemicals to facilitate their central permeation. This pathophysiological condition simplifies the CNS penetration of those phytochemicals with limitations in their penetration.

## Discussion

**FIGURE 3:**
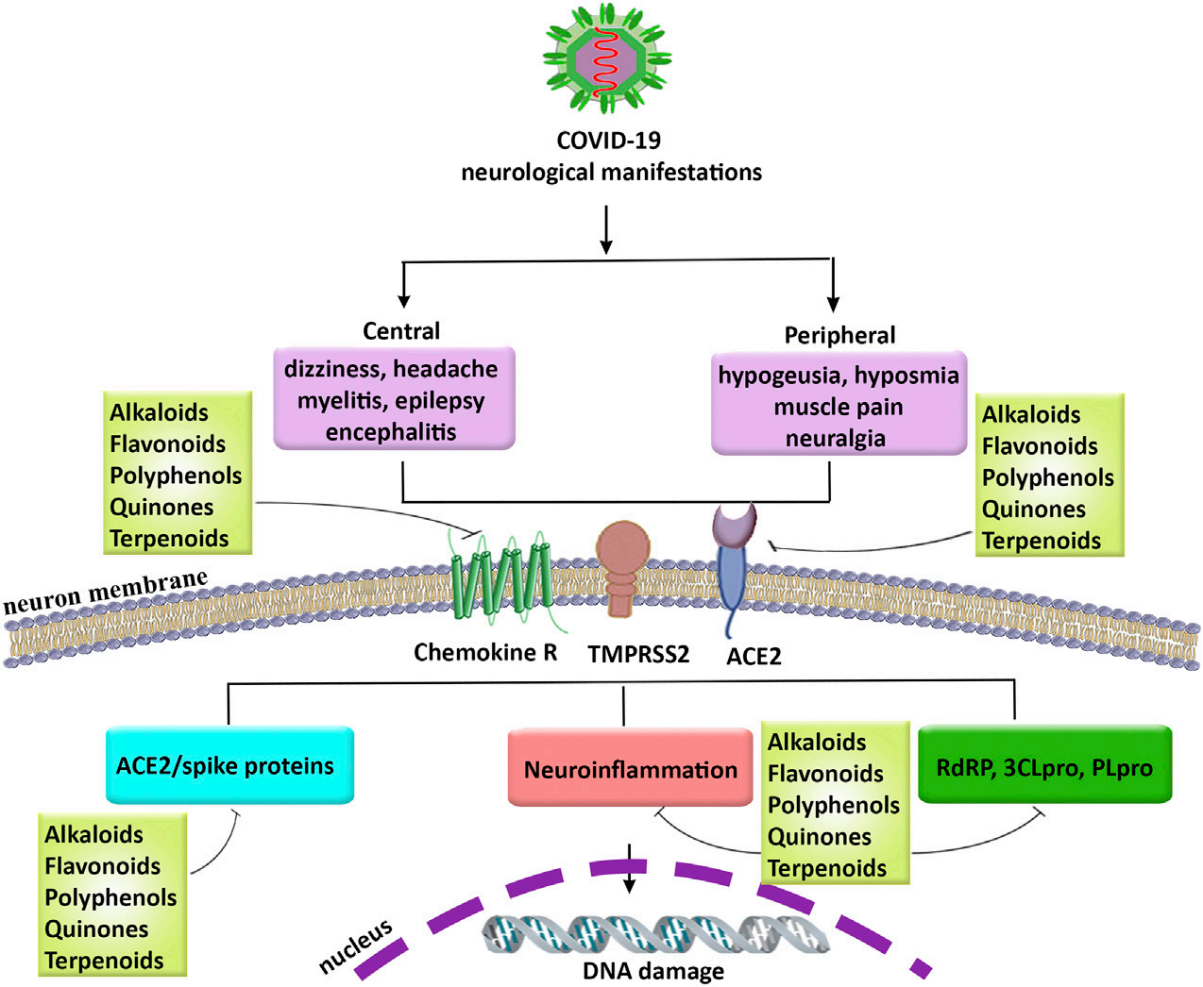
The neurological manifestations in COVID-19, related pathophysiological mechanisms, and promising role of phytochemicals. COVID-19: coronavirus 2019, PLpro: papain-like protease, RdRP: RNA-dependent RNA polymerase, SARS-CoV-2: severe acute respiratory syndrome coronavirus 2, TMPRSS2: transmembrane protease, serine 2, 3CLpro: 3-chymotrypsin-like cysteine protease.

COVID-19 pandemic is an important threat to human life. Up to now, no effective drug or vaccination has been provided to combat various complications in COVID-19. So, finding therapeutic agents to combat related manifestations in COVID-19, is of great importance. Among different complications of COVID-19 the neurological manifestations have attracted particular attention. Growing evidence is highlighting the involvement of multiple dysregulated mechanisms behind the pathophysiology of COVID-19 neurological manifestations, including hypoxia, neuroinflammation, ACE2/spike proteins, and related enzymes in virus proliferation (e.g., RdRP, 3CLpro, and PLpro). So, providing multi-target agents could pave the road in combating associated neuronal manifestations in COVID-19. For many years, the plant kingdom has shown promising antiviral, and anti-neuroinflammatory results. Accordingly, the hope regarding identifying new applications for the candidate phytochemicals has a successful history in complementary/alternative medicine. We previously showed the antiviral approaches and therapeutic targets of plant-derived secondary metabolites in various steps of viruses life cycle, including penetration, uncoating, replication, and release (Pour et al., 2019). In the present study, potential phytochemicals with antiviral effects and modulatory potentials against neuroinflammation, ACE2/spike protein, and related main proteases in the virus life cycle have been highlighted regarding inhibiting the penetration/attachment and replication phases of coronaviruses (Figure 3). Among the aforementioned phytochemicals, *in silico*/*in vitro* results introduced polyphenols (mainly flavonoids), alkaloids, and terpenes/terpenoids as potential candidates in counteracting the neurological signs of COVID-19. Although the BBB limits the CNS penetration of some phytochemicals, the disease-related inflammatory conditions as well as novel delivery systems could potentially overcome the BBB dynamic and drawback the limitation. As the results, flavonoids like naringin and it aglycone (naringenin), theaflavins, silymarin, curcumin, EGCG, polyphenol resveratrol and its derivative (polydatin), as well as some phytosterols and cannabinoids showed the most simultaneous anti-neuroinflammatory and antiviral potentials in combating SARS-CoV-2 neural complications. To suppress the viral penetration/attachment the flavonoids hesperidin, chrysin, kaempferol, quercetin, fisetin, baicalin, naringenin, EGCG, and theaflavin as well as some terpenes chalcones, glycyrrhizin, nimbin and alkaloids like berberin, thebaine, piperine as well as terpenoids have shown a more potential future in targeting ACE2/spike proteins. Consequently, regarding targeting the main proteases of coronaviruses flavonoids apigenin, cyaniding, delphinidin, EGCG, theaflavin, naringenin, hesperidin, quercetin and kaempferol, as well as some chalcones, steroidal alkaloid, terpenoids, and quinones are of potential candidates in inhibiting the main proteases of coronaviruses. Overall, the aforementioned phytochemicals have shown growing evidence to be of potential agents in combating neurological signs of COVID-19 through attenuation of neuroinflammation, ACE2/spike proteins, and main proteases.

Such studies could pave the road regarding finding novel therapeutic agents in combating neurological manifestations in COVID-19. Further reports are required to reveal the precise dysregulated pathways responsible for COVID-19 neurological signs, as well as potential therapeutic phytochemicals.
